# Mapping the Literature on Self-Efficacy Levels Among Medical Students in Higher Education: Protocol for a Scoping Review to Guide Educational Strategies

**DOI:** 10.2196/95032

**Published:** 2026-10-27

**Authors:** Alex Bertolazzo Quitério, Emerson Roberto dos Santos, João Daniel de Souza Menezes, Matheus Querino da Silva, Clarissa Albuquerque Vaz Nunes, Denise Iglesias Lima Silvestrini, Alana Clara Santos Rocha, Pedro Belchior da Silveira Junior, Andressa Karina Stefani, Thiago Sivieri, Gustavo Gouveia Macedo, Camila Bortoluci de Lima, Janaína Aparecida de Sales Floriano, Marina Ottoboni Rossi, Giovana Viecili Rossi, Ana Paula Bernardes da Rosa, Luís Cesar Fava Spessoto, Gerardo Maria de Araújo Filho, Antônio Hélio Oliani, Denise Cristina Móz Vaz, Izaura dos Santos Ramos, Fabiana de Souza Orlandi, Samila Bernardi do Vale Lopes, Julio César André

**Affiliations:** 1 Center for Studies and Development of Health Education – CEDES São José do Rio Preto Medical School - FAMERP São José do Rio Preto, São Paulo Brazil; 2 University of Beira Interior Covilhã, Castelo Branco Portugal; 3 Federal University of São Carlos São Carlos, São Paulo Brazil

**Keywords:** self-efficacy, medical students, academic performance, medical education, scoping review protocol

## Abstract

**Background:**

Self-efficacy significantly influences medical students’ academic performance. Rooted in Albert Bandura’s social cognitive theory, self-efficacy is defined as an individual’s subjective judgment of their capability to organize and execute courses of action required to attain designated types of performances. Unlike general self-esteem, self-efficacy is task- and context-specific. In undergraduate medical education, self-efficacy operates across three interdependent subdomains: (1) academic self-efficacy, encompassing cognitive confidence to master biomedical curricula; (2) clinical skills self-efficacy, reflecting perceived competence in procedural tasks and clinical reasoning; and (3) interpersonal and communication self-efficacy, denoting confidence in empathetic history taking and interprofessional collaboration. These subdomains are presented as a provisional analytical heuristic informed by Bandura’s social cognitive theory and prior reviews of self-efficacy in medical education.

**Objective:**

This scoping review aims to map and analyze the literature on self-efficacy among medical students, identifying factors in its construction and influence on academic performance. The review also aims to catalog the instruments used to measure self-efficacy and examine changes in self-efficacy over time and across stages of medical training.

**Methods:**

Following the frameworks of Arksey and O’Malley, Levac et al, and the Joanna Briggs Institute, this review addresses the question, “What has been published about self-efficacy levels in higher education among medical students?” Comprehensive searches across major electronic databases and gray literature will include studies from January 2019 to September 2026, in English, Portuguese, or Spanish, focusing on undergraduate medical students. Searches will be conducted in the Embase, Cochrane Library, PubMed, Scopus, Virtual Health Library, Web of Science databases and in the education- and psychology-focused databases ERIC and PsycInfo using an expanded search strategy incorporating controlled vocabulary (MeSH [Medical Subject Headings], Emtree, and DeCS [Descritores em Ciências da Saúde]) and free-text synonyms. Screening will be performed using Covidence software (Veritas Health Innovation), with discrepancies resolved by a third senior reviewer. Data will be narratively synthesized through descriptive qualitative mapping and structured tabular summaries, without quantitative statistical pooling.

**Results:**

This protocol was registered prospectively on the Open Science Framework before manuscript submission. This research has not received specific funding from any agency in the public, commercial, or not-for-profit sectors. No data have been collected as of September 2026. Each of the 4 review phases is estimated to take 2 months. Results are expected to be published approximately 9 to 12 months following the initiation of database searches.

**Conclusions:**

The resulting evidence map will clarify self-efficacy constructs within medical education, delineate psychometric assessment practices, and identify curricular strategies that foster clinical and academic self-efficacy, providing actionable guidance for medical educators and curriculum planners.

**Trial Registration:**

Open Science Framework 10.17605/OSF.IO/RHZDX; https://doi.org/10.17605/OSF.IO/RHZDX

**International Registered Report Identifier (IRRID):**

PRR1-10.2196/95032

## Introduction

### Background

Self-efficacy, defined by Albert Bandura’s social cognitive theory [1] as the confidence to perform the necessary activities to achieve a desired goal, emerges as an important factor influencing students’ academic performance [2]. According to this theory, the factors that influence behavior are embedded in the belief that one has the ability to perform it. In this sense, as one of the main objectives of students at all levels is to obtain good grades and a high level of academic success [3], understanding this self-confidence gains substantial relevance, especially in relation to medical education, representing a fundamental starting point for the development of effective educational strategies aimed at these students.

### Theoretical Grounding and Construct Operationalization

Rooted in Bandura’s social cognitive theory, self-efficacy is defined as an individual’s subjective judgment of their capability to organize and execute courses of action required to attain designated types of performances. Unlike general self-esteem, which represents an affective evaluation of self-worth, self-efficacy is task and context specific, acting as a direct cognitive mediator of behavioral execution, effort expenditure, and emotional resilience under duress. In undergraduate medical education, self-efficacy operates across three distinct yet interdependent subdomains: (1) academic self-efficacy, encompassing the cognitive confidence to master extensive biomedical curricula and succeed in standardized examinations; (2) clinical skills self-efficacy, reflecting perceived competence in executing procedural tasks, physical examinations, and clinical reasoning during patient encounters; and (3) interpersonal and communication self-efficacy, denoting confidence in conducting empathetic history taking, interprofessional collaboration, and delivering sensitive medical information. Conflating these subdomains with related psychological traits, such as resilience, grit, and generalized optimism, obscures the mechanistic understanding of how pedagogical interventions foster student development. This scoping review explicitly maps evidence across these 3 operationalized subdomains while cataloging the specific psychometric scales used to evaluate each construct. These three subdomains are grounded in Bandura’s 4 sources of self-efficacy (mastery experiences, vicarious experiences, verbal persuasion, and physiological or affective states) and in the domain-specificity literature [4,5]. They are proposed as a provisional analytical heuristic for charting, and the review will remain open to additional or alternative self-efficacy domains that emerge from the included literature.

Several authors have shown a correlation between self-efficacy and defining elements of academic success. Carranza et al [6] demonstrated a significant correlation with study satisfaction, whereas Hwang and Oh [7] correlated it with self-regulated learning, self-directed learning, and problem-solving ability. It should be noted that some of the supporting studies cited in this paper’s Introduction section involve nursing, paramedical, or general graduate students rather than medical students; they are cited as related educational evidence and will not be treated as direct medical education evidence in this review.

In this same context, a crucial element in all academic activities is finding the motivation to learn a chosen subject. This motivation is what “initiates and sustains” learning and can be driven by intrinsic and extrinsic factors, such as career, grades, interest, self-determination, and self-efficacy [8]. Thus, intrinsic motivation is related not only to self-efficacy but also to engagement in learning and school performance [9].

Recent studies highlight that self-efficacy not only predicts academic performance but also is fundamental in active teaching methodologies. In a 2020 study, self-efficacy was the only direct predictor of medical students’ academic performance in a flipped classroom environment, suggesting that the more strongly students believe in their ability to perform actions that help them achieve academic goals, the higher their test scores will be [10]. Moreover, Hayat et al [2] found strong predictive links between academic self-efficacy, positive emotions, metacognitive learning strategies, and academic performance, which are extremely important, as metacognitive learning, such as self-regulated learning, plays an important role in the successful application of knowledge and the acquisition of professional and clinical competence [11].

From the perspective of social education, when the educational context and the particularities of each student are understood, it becomes evident that psychological factors have a great influence on the teaching-learning process. Thus, it can be expected that when students believe in their ability to successfully perform their tasks, they enjoy the learning process more, with better feelings such as hope and pride, compared to students with low self-efficacy [2].

It has been widely suggested in the specialized literature that to improve performance among medical students, it is important to help them increase their self-efficacy [10]. Thus, not only would improvements in self-efficacy and self-esteem strengthen resilience and motivation for learning and professional development but also interventions aimed at this goal would provide positive internal feedback and help students perform difficult tasks while learning to face them in a meaningful way and with a favorable climate [12].

As psychological well-being among medical students should be among the highest priorities in the development of programs focused on educational interventions [12], promoting academic motivation and recognizing and applying the skills of managing family, professional, and university tasks, while understanding the factors that affect stress, motivation, and self-efficacy, enable students to overcome the stress of everyday life with greater interest and effort and protect themselves against academic burnout [13].

### Positioning of This Review Relative to Previous Literature

In our research, few review studies were found whose theme was self-efficacy exclusively among medical students. Costa et al [14] found limitations such as sample restriction by language and the period searched and suggested the inclusion of other databases. Visoso [15] suggested exploring regions other than the United States to understand the influences of self-efficacy and factors that influence it. The most comprehensive prior synthesis is the critical review by Klassen and Klassen [16], which examined 74 studies and identified major conceptual and measurement problems in medical student self-efficacy research, including inconsistent construct definitions and reliance on instruments of uncertain validity. The present review is explicitly positioned as an update and extension of that work, focusing on the literature published from January 2019 onward while retaining backward and forward citation searching to capture foundational studies. As an update, this review does not claim to provide a comprehensive map of the entire historical literature on medical student self-efficacy; rather, it provides a contemporary map of the field since 2019, building on and extending the previous critical review [16].

The question guiding this review and its objectives indicate the need for a scoping review rather than a systematic review [17,18]. This review should map, review, and analyze studies on academic self-efficacy among medical students, in addition to understanding factors related to its construction and influence on academic performance. The review is justified by seeking to address knowledge gaps that previous reviews were unable to address and by the lack of available scoping reviews on the topic.

### Objectives

Therefore, this scoping review will aim to (1) identify and map existing studies on self-efficacy among medical students; (2) analyze recent trends in this research area, including factors that may relate to the construction, maintenance, and changes in self-efficacy among these students; (3) identify and analyze knowledge gaps in this field, highlighting areas that require further research and investigation; (4) identify and catalog the instruments used to measure self-efficacy among medical students, including their psychometric properties and validity evidence; and (5) examine changes in self-efficacy over time and across stages of medical training.

It is expected, therefore, that this review will provide valuable insights for educators, researchers, and professionals interested in improving teaching and learning in medical education, contributing to the development of effective and sustainable strategies in this educational field.

## Methods

### Overview

This scoping review will be conducted according to the methodological frameworks proposed by Arksey and O’Malley [19] and Levac et al [20] and the recommendations of the Joanna Briggs Institute (JBI) [21]. The updated JBI guidance for scoping reviews will be used as the principal conduct framework, and PRISMA-ScR (Preferred Reporting Items for Systematic Reviews and Meta-Analyses extension for Scoping Reviews) will be used primarily as the reporting guideline for the completed review [22].

The research question guiding this review is “What has been published in the literature about the levels of self-efficacy in higher education among medical students?” This question was established using the PCC strategy (population: medical students; concept: self-efficacy; and context: academic or higher education). The PCC strategy is widely used to formulate research questions in scoping and systematic reviews [23]. The 3 subdomains described in the Introduction section will be used as a provisional analytical heuristic for charting; the review remains open to self-efficacy domains identified in the included literature, and the final charting framework will be refined iteratively during data extraction.

Inclusion and exclusion criteria were defined based on the research question and the objective of the review. Table 1 presents the inclusion and exclusion criteria in detail.

**Table 1:** Population, concept, context (PCC) eligibility framework.

PCC^a^ dimension	Inclusion criteria	Exclusion criteria
Population	Undergraduate medical students enrolled in recognized higher education institutions (preclinical, paraclinical, and clinical clerkship phases)	Postgraduate medical residents, fellows, practicing physicians, and students from non–medical health sciences
Concept	Primary or secondary investigations assessing perceived self-efficacy levels, psychometric validation of self-efficacy tools, or educational interventions targeting self-efficacy enhancement	Studies examining general psychological distress, burnout, or resilience without explicit, isolated measurement or conceptualization of self-efficacy
Context	Undergraduate medical education settings worldwide, including university classrooms, simulation laboratories, hospital wards, and community ambulatory clinics	Nonacademic settings, commercial coaching environments, or continuing professional medical development programs
Study types and sources	Quantitative, qualitative, and mixed methods empirical studies; systematic reviews; observational cohort, cross-sectional, and experimental and quasi-experimental trials published between January 2019 and January 2026 in English, Portuguese, or Spanish	Editorials, opinion pieces, conference abstracts without full text, book chapters, non–peer-reviewed commentaries, and studies published prior to 2019

^a^PCC: population, concept, context.

Systematic reviews and meta-analyses will be included as contextual material and as sources for citation searching; they will be charted separately and will not be treated as primary evidence for self-efficacy levels, instruments, or intervention effects. Primary studies will constitute the unit of analysis for empirical findings.

### Search Strategy and Information Sources

#### Search Timeframe Rationale

The restriction to January 2019 to September 2026 is grounded in two transformative shifts in medical education: (1) the worldwide overhaul of undergraduate medical curricula toward competency-based medical education frameworks following global accreditation mandates and (2) the unprecedented disruptions, hybrid pedagogical adaptations, and psychological stressors introduced during and following the COVID-19 pandemic, which redefined medical student self-efficacy paradigms. To mitigate the risk of omitting foundational evidence, backward and forward citation searching (pearling) of all included studies will be conducted without date restrictions. The search will be updated immediately before the final data analysis to ensure that the evidence base is current at the time of completion.

Searches will be conducted in the following electronic databases: Embase (Elsevier), Cochrane Library (Wiley), PubMed (National Library of Medicine), Scopus (Elsevier), Virtual Health Library (BIREME, Pan American Health Organization), and Web of Science (Clarivate), as well as in ERIC (Institute of Education Sciences) and PsycInfo (American Psychological Association). Additionally, a search for gray literature will be conducted, including theses, dissertations, government reports, and conference proceedings. ERIC and PsycInfo were added because of their direct relevance to the educational and psychological focus of this review; the Cochrane Library will be searched but is expected to yield limited results for this topic. Gray literature searching will include ProQuest Dissertations & Theses Global (Clarivate), conference proceedings (eg, AMEE and Ottawa Conference abstracts), OpenGrey, and a targeted Google Scholar (Google LLC) search (first 200 results screened). Detailed strategies for these sources are provided in Multimedia Appendix 1.

The search strategy was developed in collaboration with a medical research librarian and incorporated controlled vocabulary (MeSH [Medical Subject Headings], Emtree, and DeCS [Descritores em Ciências da Saúde]) and extensive free-text synonyms covering the following: (1) self-efficacy and related cognitive appraisals (eg, “self-efficacy,” “perceived competence,” “academic confidence,” and “clinical self-efficacy”), (2) medical students and undergraduate medical education (eg, “medical student,” “undergraduate medical education,” “clinical clerk,” and “trainee physician*”), and (3) higher education and curriculum settings. The detailed search strategy adapted for each database is provided in Multimedia Appendix 1.

To ensure accuracy and comprehensiveness in the search, the search strategy will follow the 3-step approach recommended by the JBI guidance on the conduct of narrative synthesis in systematic reviews [24]. The 3-step search approach is formally recommended by the *JBI Manual for Evidence Synthesis* [22,25]; the previous citation to narrative synthesis guidance has been corrected.

#### Initial Limited Search

An initial limited search will be conducted in selected databases (eg, PubMed and Scopus) to identify initial relevant articles, analyze their keywords, and refine subject headings or index terms (MeSH for PubMed and Emtree for Embase).

#### Comprehensive Search

A second, comprehensive search will then be performed across all listed electronic databases and gray literature sources using all identified keywords and index terms. The descriptors used in the search were “Self-efficacy” and “Students, Medical” combined with Boolean operators. All descriptor management processes were carried out with the guidance of a librarian specializing in the health field. The search strategy used the descriptors associated with the Boolean operators AND and OR and was customized for each of the databases. The detailed search strategy adapted for each database is provided in Multimedia Appendix 1.

#### Reference List Searching

The reference lists of all studies selected for full-text review will be manually screened to identify additional relevant articles.

### Study Selection and Screening Process

The study selection process will be performed by 2 reviewers independently, using the Covidence (Veritas Health Innovation) reference management software [26]. Two independent reviewers (JDdSM and ABQ) will perform title and abstract screening, followed by full-text assessment. Discrepancies will be resolved by consensus or by a third senior reviewer (GMdAF). Data extraction will be performed by 2 independent reviewers (MQdS and CAVN) using a standardized form. The third senior reviewer will adjudicate any extraction discrepancies. The selection process will follow the recommendations of PRISMA-ScR [5] and will be presented through a flowchart. A PRISMA-ScR flowchart template is provided in Multimedia Appendix 2 [5] and will be completed with the actual numbers of records identified, screened, excluded, and included in the Results section of the final review.

Data will be extracted using a standardized form, developed specifically for this review [25]. The form will include information on authorship, year of publication, country, objective, study design, population, outcomes assessed, main results, and conclusions. The extraction form will additionally capture the definition and domain of self-efficacy used by each study, instrument validity evidence and scoring, intervention content and dose, comparator, assessment timing, and effect estimates, where reported. A preliminary draft of the data extraction form is presented in Multimedia Appendix 3.

### Data Extraction, Charting, and Synthesis

Data synthesis will be performed narratively, with tables and graphs to summarize the findings [27]. For the analysis of extracted data, a conceptual mapping approach will be used, where information will be grouped into emergent categories related to factors influencing self-efficacy, academic trends, and knowledge gaps. Qualitative analyses, such as thematic analysis, may be used to identify recurring themes in the included studies [28]. Thematic analysis will be used to identify recurring themes in the included studies. In accordance with JBI guidelines for scoping reviews, no statistical pooling, effect size aggregation, or quantitative meta-analysis will be conducted. Instead, data will be synthesized through descriptive qualitative mapping and structured tabular summaries. Data charting will categorize evidence across three analytical axes: (1) epidemiological and curricular mapping, which involves distribution of studies across geographic regions, institution types, and training stages (preclinical vs clinical clerkship years); (2) instrument characterization, which involves cataloging of self-efficacy psychometric scales, distinguishing between validated generalized scales (eg, General Self-Efficacy Scale), adapted domain-specific instruments, and custom ad-hoc questionnaires; and (3) pedagogical interventions, which involves taxonomic grouping of educational strategies (eg, high-fidelity simulation, deliberate clinical practice, peer-assisted learning, flipped classroom models, and mindfulness and psychological skills training) evaluated for their capacity to enhance self-efficacy. Conceptual gaps, measurement inconsistencies, and geographic disparities will be highlighted using descriptive evidence maps. Because scoping reviews do not include formal critical appraisal of individual sources of evidence, this review will map intervention findings but will not recommend specific educational strategies or infer intervention effectiveness.

This scoping review will follow the recommendations of PRISMA-ScR [4,5] to ensure transparency and quality of reporting. The respective protocol for this review was developed considering the PRISMA-P (Preferred Reporting Items for Systematic Reviews and Meta-Analyses Protocols) checklist and has been registered with the Open Science Framework prior to manuscript submission (DOI: 10.17605/OSF.IO/RHZDX) to promote transparency and reproducibility of the review process [29].

### Consultation With Experts

A structured consultation with experts will be conducted after the preliminary synthesis of data. This step aims to validate the preliminary findings and discuss the interpretation of results and their implications for the development of educational strategies in medical education. This consultation may involve medical educators, psychologists, or other relevant stakeholders.

### Expected Outcomes of the Scoping Review

The anticipated outcomes described below are exploratory hypotheses and possible outputs; they do not constitute predictions of results and will be confirmed, refuted, or refined against the mapped evidence.

This scoping review, by systematically mapping and analyzing the literature on self-efficacy among medical students, is anticipated to generate a comprehensive and valuable set of outcomes for medical education.

First, it is expected to provide a detailed and up-to-date overview of the existing research on self-efficacy among medical students. This will encompass the identification of publication volumes, predominant methodological approaches used, geographical regions most active in research, and the demographic characteristics of study participants. Ultimately, a clear understanding of “who is researching what and where” concerning this vital concept will be established.

Second, the review is poised to analyze recent trends and the various factors associated with the construction, maintenance, and potential fluctuations in self-efficacy among these students. This may include the identification of key determinants, which includes the principal predictors and correlates of self-efficacy, such as prior academic performance, faculty feedback, practical experiences, social support, and personality traits; educational contexts, which refers to how diverse teaching methodologies (eg, problem-based learning, flipped classroom, and clinical simulation) and learning environments (online versus in person) impact the development of self-efficacy; and challenges and enablers, which refer to the obstacles encountered and the strategies used by both students and educational institutions to foster self-efficacy throughout medical training.

Third, the review will be pivotal in identifying knowledge gaps and areas warranting further investigation. By consolidating existing research, we will be able to pinpoint aspects of self-efficacy in medical students that remain underexplored, such as the influence of cultural and socioeconomic factors, the impact of specific mentorship programs, self-efficacy across different stages of medical training (eg, clinical vs preclinical years), and the effectiveness of interventions specifically designed to bolster self-efficacy. This identification of gaps will serve as an essential guide for future research, directing the scholarly agenda in the field.

Finally, the findings of this review will have direct practical applications for enhancing teaching and learning in medical education. By gaining a deeper understanding of how self-efficacy is constructed and influenced, medical educators and program administrators will be empowered to develop more effective pedagogical strategies by adapting curricula and methodologies to cultivate self-efficacy as a cornerstone of academic and professional success; implement targeted interventions through the creation of psycho-pedagogical support programs that address the specific needs of students, thereby promoting greater self-confidence and resilience; and optimize academic performance and well-being to ensure that future health care professionals not only achieve academic success but also develop a proactive, motivated, and self-assured attitude, which is indispensable for medical practice.

In summary, this scoping review will not only systematize existing knowledge but also serve as a fundamental resource to inform evidence-based educational practices and catalyze novel investigations aimed at optimizing the development of self-efficacy in medical students.

## Results

As of September 2026, no data have been collected for this review. This research has not received specific funding from any agency in the public, commercial, or not-for-profit sectors. The protocol was registered prospectively on the Open Science Framework prior to manuscript submission. Electronic database searches are projected to commence in October 2026, following protocol acceptance, with an estimated 2 months for the comprehensive literature search, 2 months for study selection, 2 months for data extraction, and 2 months for data analysis and synthesis. Final results are expected to be published in the second half of 2027. A PRISMA-ScR flowchart will be completed, with the actual numbers of records identified, screened, excluded, and included, and will be presented in the Results section of the final review; a template is provided in Multimedia Appendix 2.

## Discussion

### Principal Considerations and Anticipated Findings

Medical students confront unique stressors that directly challenge their psychological well-being and academic progression. Self-efficacy represents a critical cognitive mechanism that determines whether a trainee approaches complex clinical scenarios as challenges to be mastered or threats to be avoided. By systematically charting the literature from 2019 to 2026, this scoping review will provide the first unified synthesis of how self-efficacy is defined, measured, and cultivated across contemporary undergraduate medical curricula. The anticipated findings described below are exploratory hypotheses to be tested against the mapped evidence; they do not constitute predictions of results. We anticipate revealing substantial heterogeneity in psychometric measurement, with a disproportionate reliance on unvalidated, self-administered questionnaires at the expense of standardized, domain-specific instruments. Without a formal critical appraisal, this review may map intervention findings but will not recommend specific educational strategies or infer intervention effectiveness.

### Comparison With Prior Work

Prior reviews in health professions education have primarily evaluated burnout, anxiety, or broad psychological resilience, treating self-efficacy as a secondary or incidental covariate. When self-efficacy has been investigated, reviews have largely grouped all health care disciplines together, combining medical, nursing, and allied health trainees, thereby obscuring the specific structural, cultural, and pedagogical dynamics of medical school training. This scoping review fills this gap by focusing strictly on medical students and delineating self-efficacy trajectories across distinct educational phases, contrasting preclinical foundational learning with immersive hospital-based clerkships. In doing so, it builds directly on the critical review by Klassen and Klassen [16], which identified persistent conceptual and measurement problems in this literature and called for more rigorous, domain-specific research; the present review will assess the extent to which those problems persist in the 2019 to 2026 literature.

### Strengths and Limitations

The strengths of this protocol include adherence to rigorous JBI and PRISMA-ScR guidelines, preregistration on the Open Science Framework, a comprehensive search architecture across 6 prominent biomedical databases without geographic restrictions, dual independent screening and data extraction protocols using Covidence, and trilingual coverage (English, Portuguese, and Spanish). Limitations include restricting the primary search time frame to 2019 to 2026, which, although justified by recent curriculum reforms and postpandemic adaptations, risks omitting earlier foundational studies. To mitigate this, backward citation searching will be executed on all included studies. As an update of the earlier critical review by Klassen and Klassen [16], this review is explicitly framed as a contemporary map of the field since 2019 rather than a comprehensive historical map. Furthermore, scoping reviews do not mandate formal risk-of-bias assessment, meaning that the overall methodological quality of primary studies will not be quantitatively weighted.

### Dissemination Plan

In accordance with *JMIR Research Protocols* standards, the findings of this review will be actively disseminated through multiple scholarly and operational channels: (1) publication of the completed review in an open-access peer-reviewed medical education journal; (2) presentation at national and international medical education conferences (eg, AMEE and Ottawa Conference); and (3) translation into an open-access executive summary distributed to medical school curriculum committees, deans of medical education, and student mental health task forces to inform evidence-based pedagogical design.

The summary of the current literature through this scoping review will aid in understanding not only self-efficacy among medical students but also which factors are associated with its construction. Furthermore, knowing the self-perception of students throughout their academic trajectory in higher education may serve as support in the development of new teaching strategies and adaptation of the current model. Thus, this study has practical applications in the teaching-learning context of the training of new medical professionals, which may reflect in better academic performance of these professionals and the development of modern curricula adapted to contemporary times.

### Conclusions

This protocol for a scoping review establishes a rigorous methodological foundation to clarify how self-efficacy is conceptualized, quantified, and enhanced in undergraduate medical education. The resulting evidence map will serve as an essential resource for educational leaders seeking to design targeted interventions that bolster student confidence, clinical competence, and professional identity formation. The conclusions drawn will be strictly bounded by the descriptive, mapping nature of the scoping review methodology.

This protocol meticulously outlines a robust and systematic approach to conducting a scoping review on self-efficacy among medical students. By adhering to established methodological frameworks and incorporating rigorous search, selection, and data extraction strategies, this protocol ensures transparency and reproducibility. Ultimately, this detailed roadmap will enable a comprehensive mapping of existing literature and the identification of crucial knowledge gaps, thereby providing valuable evidence to inform and enhance educational strategies aimed at fostering self-efficacy and optimizing academic outcomes in medical education.

## Appendix

Multimedia Appendix 1 Customized search strategy for each database.

Multimedia Appendix 2 PRISMA-ScR flowchart template (to be completed in the Results section of the final review).

Multimedia Appendix 3 Preliminary data extraction form.

## Abbreviations

DeCS: Descritores em Ciências da Saúde
JBI: Joanna Briggs Institute
MeSH: Medical Subject Headings
PCC: population, concept, context
PRISMA-ScR: Preferred Reporting Items for Systematic Reviews and Meta-Analyses extension for Scoping Reviews

## Data Availability

All data generated or analyzed during this study will be included in the published scoping review article.
