# Supplementary material for: Mapping the Literature on Self-Efficacy Levels Among Medical Students in Higher Education: Protocol for a Scoping Review to Guide Educational Strategies
Source: JMIR Res Protoc. 2026 Oct 27;15:e95032. doi: 10.2196/95032 (PMC13752587; doi:10.2196/95032)
Supplement: Supplementary file 1 [file resprot_v15i1e95032_app1.docx]

Multimedia Appendix 1. Customized search strategy for each database

| **Database** | **Search Query String** |
| --- | --- |
| PubMed | ("Self Efficacy"[Mesh] OR "self-efficacy"[Title/Abstract] OR "perceived competence"[Title/Abstract] OR "academic confidence"[Title/Abstract] OR "clinical self-efficacy"[Title/Abstract]) AND ("Students, Medical"[Mesh] OR "medical student"[Title/Abstract] OR "undergraduate medical education"[Title/Abstract] OR "clinical clerk"[Title/Abstract] OR "trainee physician"[Title/Abstract]) **Filters: 2019/01/01 to 2026/09/30, English, Portuguese, Spanish. [The third concept (higher education or curriculum) was removed to maximize sensitivity; the context is confirmed at screening.]** |
| Embase | ***('self efficacy'/exp OR 'self-efficacy':ti,ab OR 'perceived competence':ti,ab OR 'academic confidence':ti,ab OR 'clinical self-efficacy':ti,ab) AND ('medical student'/exp OR 'medical student':ti,ab OR 'undergraduate medical student':ti,ab OR 'clinical clerk':ti,ab) AND [2019-2026]/py. [Additional Emtree terms added; third concept removed.]*** |
| Cochrane Library | ("self-efficacy" OR "perceived competence" OR "academic confidence") AND ("medical student" OR "undergraduate medical education" OR "clinical clerk") with Publication Year from 2019 to 2026 **in Title Abstract Keyword. [Third concept removed.]** |
| Scopus | TITLE-ABS-KEY(("self-efficacy" OR "perceived competence" OR "academic confidence" OR "clinical self-efficacy") AND ("medical student" OR "undergraduate medical education" OR "clinical clerk")) AND PUBYEAR > 2018 AND PUBYEAR < 2027 **AND LANGUAGE(English OR Portuguese OR Spanish). [Third concept removed.]** |
| Virtual Health Library (VHL, DeCS) | (mh:("Autoeficácia" OR "Self Efficacy" OR "Autoeficacia") OR ti:("self-efficacy" OR "autoeficácia" OR "autoeficacia")) AND (mh:("Estudantes de Medicina" OR "Students, Medical" OR "Estudiantes de Medicina") OR ti:("medical student" OR "estudante de medicina" OR "estudiante de medicina")) **AND (year_cluster:[2019 TO 2026]) AND (la:("en" OR "pt" OR "es")). [Third concept removed; additional DeCS synonyms added.]** |
| Web of Science | TS=(("self-efficacy" OR "perceived competence" OR "academic confidence" OR "clinical self-efficacy") AND ("medical student" OR "undergraduate medical education" OR "clinical clerk")) Timespan: 2019-01-01 to 2026-09-30 **and Languages: English, Portuguese, Spanish. [Third concept removed.]** |
| **ERIC (EBSCOhost)** | ***S1: DE "Self Efficacy" OR TI "self-efficacy" OR AB "self-efficacy" OR TI "perceived competence" OR AB "perceived competence" OR TI "academic confidence" OR AB "academic confidence"; S2: DE "Medical Students" OR TI "medical student" OR AB "medical student" OR TI "undergraduate medical education" OR AB "undergraduate medical education" OR TI "clinical clerk" OR AB "clinical clerk"; S3: S1 AND S2; Limiters: Published Date 20190101-20260930; Language: English, Portuguese, Spanish.*** |
| **PsycInfo (EBSCOhost)** | ***S1: DE "Self-Efficacy" OR TI "self-efficacy" OR AB "self-efficacy" OR TI "perceived competence" OR AB "perceived competence" OR TI "academic confidence" OR AB "academic confidence"; S2: DE "Medical Students" OR TI "medical student" OR AB "medical student" OR TI "undergraduate medical education" OR AB "undergraduate medical education" OR TI "clinical clerkship" OR AB "clinical clerk"; S3: S1 AND S2; Limiters: Published Date 2019-2026; Language: English, Portuguese, Spanish.*** |
| **Gray literature** | **ProQuest Dissertations & Theses Global: noft("self-efficacy" OR "perceived competence" OR "academic confidence") AND noft("medical student*" OR "undergraduate medical education"), 2019-2026; OpenGrey: "self-efficacy" AND "medical student"; Google Scholar: "self-efficacy" AND "medical students" (first 200 results screened); conference proceedings (AMEE, Ottawa Conference) via conference websites and databases.** |
