# Supplementary material for: Mapping the Literature on Self-Efficacy Levels Among Medical Students in Higher Education: Protocol for a Scoping Review to Guide Educational Strategies
Source: JMIR Res Protoc. 2026 Oct 27;15:e95032. doi: 10.2196/95032 (PMC13752587; doi:10.2196/95032)
Supplement: Supplementary file 2 [file resprot_v15i1e95032_app2.docx]

Multimedia Appendix 2. PRISMA-ScR (Preferred Reporting Items for Systematic Reviews and Meta-Analyses extension for Scoping Reviews) flowchart template (to be completed in the Results section of the final review)

| **Stage** | **Description** | **Count** |
| --- | --- | --- |
| **Identification** | **Records identified from databases (PubMed, Embase, Scopus, Web of Science, Cochrane Library, Virtual Health Library, ERIC, PsycInfo) (n = __); Records identified from gray literature (n = __); Records after duplicates removed (n = __)** | **To be completed** |
| **Screening** | **Records screened (title and abstract) (n = __); Records excluded (n = __); Reports sought for retrieval (n = __); Reports not retrieved (n = __); Reports assessed for eligibility (full text) (n = __); Reports excluded with reasons (n = __)** | **To be completed** |
| **Included** | **Studies included in the scoping review (n = __); Reports of included studies (n = __)** | **To be completed** |

**Note:** This template follows the PRISMA-ScR reporting structure [1] and will be completed with the actual numbers of records identified, screened, excluded, and included once the searches and selection process are finalized.

**Reference**

1. Tricco AC, Lillie E, Zarin W, O’Brien KK, Colquhoun H, Levac D, et al. PRISMA Extension for Scoping Reviews (PRISMA-ScR): checklist and explanation. Ann Intern Med. 2018 Oct 2;169(7):467-473. [doi: 10.7326/M18-0850]
