# Supplementary material for: Mapping the Literature on Self-Efficacy Levels Among Medical Students in Higher Education: Protocol for a Scoping Review to Guide Educational Strategies
Source: JMIR Res Protoc. 2026 Oct 27;15:e95032. doi: 10.2196/95032 (PMC13752587; doi:10.2196/95032)
Supplement: Supplementary file 3 [file resprot_v15i1e95032_app3.docx]

Multimedia Appendix 3. Preliminary data extraction form

| **Category** | **Data Field** | **Description** |
| --- | --- | --- |
| Bibliographic Information | Study Number | Internal identification number for the study. |
| Bibliographic Information | Authors | First author and corresponding author. |
| Bibliographic Information | Year of Publication | Year the study was published. |
| Bibliographic Information | Journal or source | Name of the journal or publication where the study appeared. |
| Bibliographic Information | Country of Origin | Country where the study was conducted. |
| Study Characteristics | Study Objective | Primary objective(s) as stated by the authors. |
| Study Characteristics | Study Design | Methodological approach (eg, cross-sectional, longitudinal, qualitative, quantitative, mixed methods). |
| Population Studied | Total Sample Size (N) | Total number of participants in the study. |
| Population Studied | Participant Demographics | Age range or mean, gender distribution, year of medical school, academic stage. |
| Population Studied | Medical Program Type | eg, traditional, problem-based learning, integrated curriculum. |
| Self-Efficacy Assessment | Self-Efficacy Definition Used | How "self-efficacy" was defined by the study authors. |
| Self-Efficacy Assessment | Assessment Instrument(s) | Name(s) of the self-efficacy scale(s) or method(s) used (eg, General Self-Efficacy Scale, specific academic self-efficacy scale). |
| Self-Efficacy Assessment | Specific Domain of Self-Efficacy | (eg, academic, clinical skills, learning, coping, specific subject). |
| **Self-Efficacy Assessment** | **Instrument Validity Evidence** | **Psychometric properties reported by the study (eg, reliability coefficients, validity evidence, prior validation studies).** |
| **Self-Efficacy Assessment** | **Instrument Scoring** | **Scoring method, response scale, and interpretation of scores.** |
| Contextual Information | Educational Setting | (eg, classroom, simulation lab, clinical rotation, online learning). |
| Contextual Information | Intervention (if any) | Description of any educational intervention aimed at influencing self-efficacy. |
| Contextual Information | Comparator (if used) | Description of the control or comparison group or condition. |
| **Contextual Information** | **Intervention Content and Dose** | **Description of intervention components, duration, frequency, and intensity.** |
| **Contextual Information** | **Assessment Timing** | **Time points at which self-efficacy was measured (eg, baseline, post-intervention, follow-up).** |
| Key Findings | Main Results (Quantitative) | Key numerical findings related to self-efficacy (eg, mean scores, correlations with academic performance, motivation, wellbeing; statistical significance). |
| Key Findings | Main Results (Qualitative or thematic) | Key themes or concepts identified regarding the construction or influence of self-efficacy. |
| Key Findings | Factors Influencing Self-Efficacy | Identified factors (eg, past performance, vicarious experiences, verbal persuasion, physiological states). |
| **Key Findings** | **Effect Estimates** | **Reported effect sizes, mean differences, or other quantitative estimates where available.** |
| Conclusions and Implications | Study Conclusions | Main conclusions drawn by the authors. |
| Conclusions and Implications | Reported Limitations | Limitations of the study as acknowledged by the authors. |
| Conclusions and Implications | Recommendations for Future Research or Practice | Suggestions for future studies or practical applications. |
